# Physicomechanical Properties of a Technical Polyethylene Glycol-Silica-Based Hydrogel as an Alternative Low Shrinkage Dental Polymer Composite

**DOI:** 10.1021/acsomega.6c02113

**Published:** 2026-07-28

**Authors:** Abdul Fattah Nongman, Mohd Firdaus Yhaya, Norhafizah Saari, Mariatti Jaafar, Nor Aidaniza Abdul Muttlib, Matheel Zohair Yousif Al-Rawas

**Affiliations:** † Biomaterials Synthesis Laboratory (BSL), School of Dental Sciences, Health Campus, 26689 Universiti Sains Malaysia, Kubang Kerian 16150, Kelantan, Malaysia; ‡ Department of Microbiology and Parasitology, School of Medical Sciences, Health Campus, Universiti Sains Malaysia, Kubang Kerian 16150, Kelantan, Malaysia; § Polymer Engineering Program, School of Material and Mineral Resources Engineering, Engineering Campus, Universiti Sains Malaysia, Seberang Perai Selatan 14300, Penang, Malaysia; ∥ Prosthodontic Unit, School of Dental Sciences, Health Campus, Universiti Sains Malaysia, Kubang Kerian 16150, Kelantan, Malaysia

## Abstract

Dental composite resins are widely used in restorative dentistry; however, polymerization shrinkage leads to marginal gaps and reduced bond strength. An expandable hydrogel monomer can reduce shrinkage, but it may lack sufficient mechanical strength when used alone. This study developed a low-shrinkage dental composite by incorporating an expandable hydrogel monomer to counteract volumetric shrinkage after curing, aiming to maintain strong bonding to tooth structure. Polyethylene glycol 400 (PEG 400) was chemically modified with acryloyl chloride to produce diacrylated polyethylene glycol 400 (DAPEG 400) expandable hydrogels, which was formulated with silica filler loadings by weight. Statistical analysis involved the Shapiro–Wilk test and one-way ANOVA. A synthesized expandable monomer with good polymerization shrinkage properties and the incorporation of silica filler significantly enhanced the mechanical properties compared to pure cured DAPEG 400, with flexural and compressive strengths reaching 10.27 MPa (standard deviation (SD) = 0.96) and 441.42 MPa (SD = 2.80), respectively. The composite also demonstrated improved dimensional stability, showing 475.32 μg/mm^3^ (SD = 22.76) reduction in water absorption and 33.37% (SD = 2.26) decrease in volumetric swelling. Most notably, the optimal formulation containing 60% filler exhibited minimal polymerization shrinkage as low as 1.58% (SD = 0.24). The hydrogel’s water absorption and expansion after curing offset polymerization contraction, while silica filler regulates expansion to prevent cracking.

## Graphical abstract

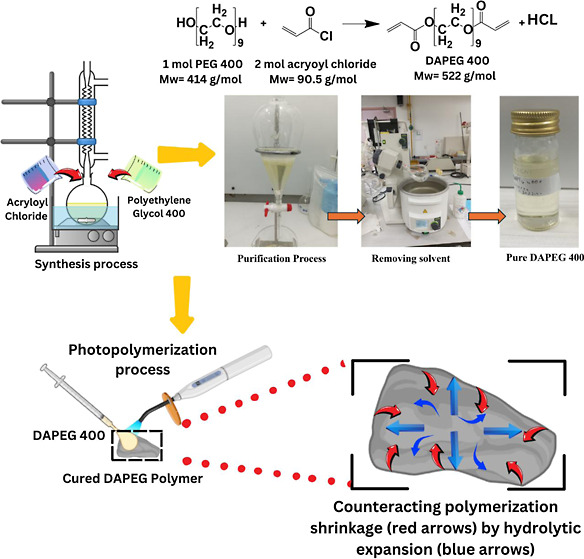

## 1 Introduction

Polymeric dental composites are favored for restorations due to their aesthetic appeal, mechanical strength, and biocompatibility.^1^ Bis-GMA (Bisphenol A-glycidyl methacrylate) is a widely used monomer in dental resins and composites due to its excellent mechanical properties and biocompatibility. However, its high molecular weight makes it difficult to handle, and it undergoes polymerization shrinkage, typically 5–7% ^2,3^ by volume upon curing as van der Waals distances between monomers collapse into covalent bonds.^4^ This shrinkage can lead to marginal gaps, secondary caries, and restoration failure.^5^ To improve handling and reduce shrinkage, diluent monomers like TEGDMA (triethylene glycol dimethacrylate) and UDMA (Urethane dimethacrylate) are added. While TEGDMA alone exhibits higher shrinkage (∼12–14%), optimized Bis-GMA/TEGDMA or BisGMA/UDMA blends can lower net shrinkage to ∼2–6%, depending on the monomer ratio and through the incorporation of fillers and also help to enhance mechanical properties.^6,7^

Recent studies have also focused on the development of new monomers to reduce polymerization shrinkage due to their larger molecular volume and ability to form cross-linked networks.^6,8,9^ Known low-shrink dental composites currently on the market operate primarily through two types of monomer: first, by utilizing monomers that undergo ring-opening polymerization (silorane monomer-based),^10,11^ and second, by incorporating acrylated monomers that exhibit both rigid and flexible characteristics (expandable monomer).^12^ These advanced monomer systems contribute to reduced polymerization shrinkage, enhancing the material’s performance. However, some low-shrinkage composite systems involve more complex monomer design and formulation, which may influence production cost depending on the brand, composition, and manufacturing process.^13,14^ Furthermore, some low shrinkage siloranes monomer-based have been discontinued, limiting the availability of this material for future research and clinical applications.^15^

Hydrogel-based monomers, known for their hydrophilic and swelling properties, have gained attention in biomedical applications.^16^ Due to their hydrophilic structure, hydrogel-based monomers can absorb water and undergo controlled expansion, which may provide a potential mechanism for postcuring shrinkage compensation.^17^ However, to ensure clinical viability, such monomers must also exhibit sufficient mechanical strength and compatibility with reinforcing fillers. Herein, polyethylene glycol (PEG) is explored as a candidate monomer for shrinkage-compensating dental composites because of its hydrophilic structure, relatively low cost, and reported biocompatibility in biomedical applications.^18–20^ The controlled expansion of PEG-based networks may help compensate for shrinkage-related dimensional changes; however, its ability to reduce marginal gap formation or secondary caries requires further validation using standardized dental testing models.^21^ Modified forms of PEG have shown potential in reducing polymerization shrinkage^17^ while preserving selected physicomechanical properties, although their performance remains dependent on formulation and testing conditions, making them a promising alternative to TEGDMA (triethylene glycol dimethacrylate) as a reactive diluent in dental composite.^22,23^ The polymerization efficiency of DAPEG has been previously reported, demonstrating high gel content and degree of conversion, which supports its suitability for composite applications.^17^ Furthermore, the strategic incorporation of silica fillers simultaneously enhances mechanical properties and reduces polymerization shrinkage in dental composites.^24,25^ As filler content increases, the relative volume of shrinkable resin matrix decreases proportionally, resulting in significantly lower overall contraction while improving composite rigidity and fracture resistance.^24^

This study focuses on the diacrylated polyethylene glycol 400 (DAPEG 400) as an expandable hydrogel monomer for dental materials, leveraging its hydrophilic polyether backbone to induce postcure expansion and counteract polymerization shrinkage. By engineering filler–matrix interactions with precisely controlled silica loadings, we propose creating a restorative material that clinically mitigates microleakage risks while maintaining the mechanical integrity required for posterior restorations. This approach offers a potentially cost-effective alternative to existing low-shrinkage systems and introduces a new direction for future dental-material innovations. It is hypothesized that the incorporation of DAPEG 400 into dental composites will result in reduced polymerization shrinkage because of its expandability in the oral environment. It was hypothesized that DAPEG 400, together with controlled silica filler loading, could reduce polymerization shrinkage while maintaining selected physicomechanical properties under in vitro conditions. At this stage, the proposed advantages of the DAPEG 400 with a silica system are considered preliminary and should not be interpreted as evidence of clinical or commercial superiority over existing dental restorative composites, as no commercial control material was evaluated under identical experimental conditions.

## 2 Materials and Methods

### 2.1 Synthesis of Silica Filler from TEOS

Silica filler was synthesized from tetraethyl orthosilicate (TEOS, ≥99.5%, for synthesis, Merck, Germany) by using the sol–gel method.^26^ Silica was synthesized by mixing 10 mL of TEOS with 60 mL of ethanol absolute (≥99.9%, Merck, Germany) and sonicating for 10 min using a 3510R-DTH sonicator (Bransonic, Mexico) operating at 40 kHz and 130 W without heating to ensure homogeneity. Then, 2 mL of distilled water was added and sonicated for 1.5 h). After adding 4 mL of ammonia solution (25%, Merck, Germany), the mixture underwent a further 3 h sonication to promote gelation, followed by 1 h of rest. The gel-like precipitate was separated by centrifugation at 7000 rpm for 5 min using a Eppendorf-5804 centrifuge (Eppendorf, Germany), then washed with acetone (≥99.8%, Merck, Germany) and distilled water, and then dried at 80 °C overnight. The dried sample was ground and calcined in a Wisetherm FH-14 furnace (Daihan, Japan) at 600 °C. at 600 °C for 30 min. The resulting silica particles ranged from 200 to 500 nm (Figure 1). Silanization was omitted, as the DAPEG resin’s CH_2_CH_2_O units enhance compatibility with the hydrophilic silica.

**Figure 1:**
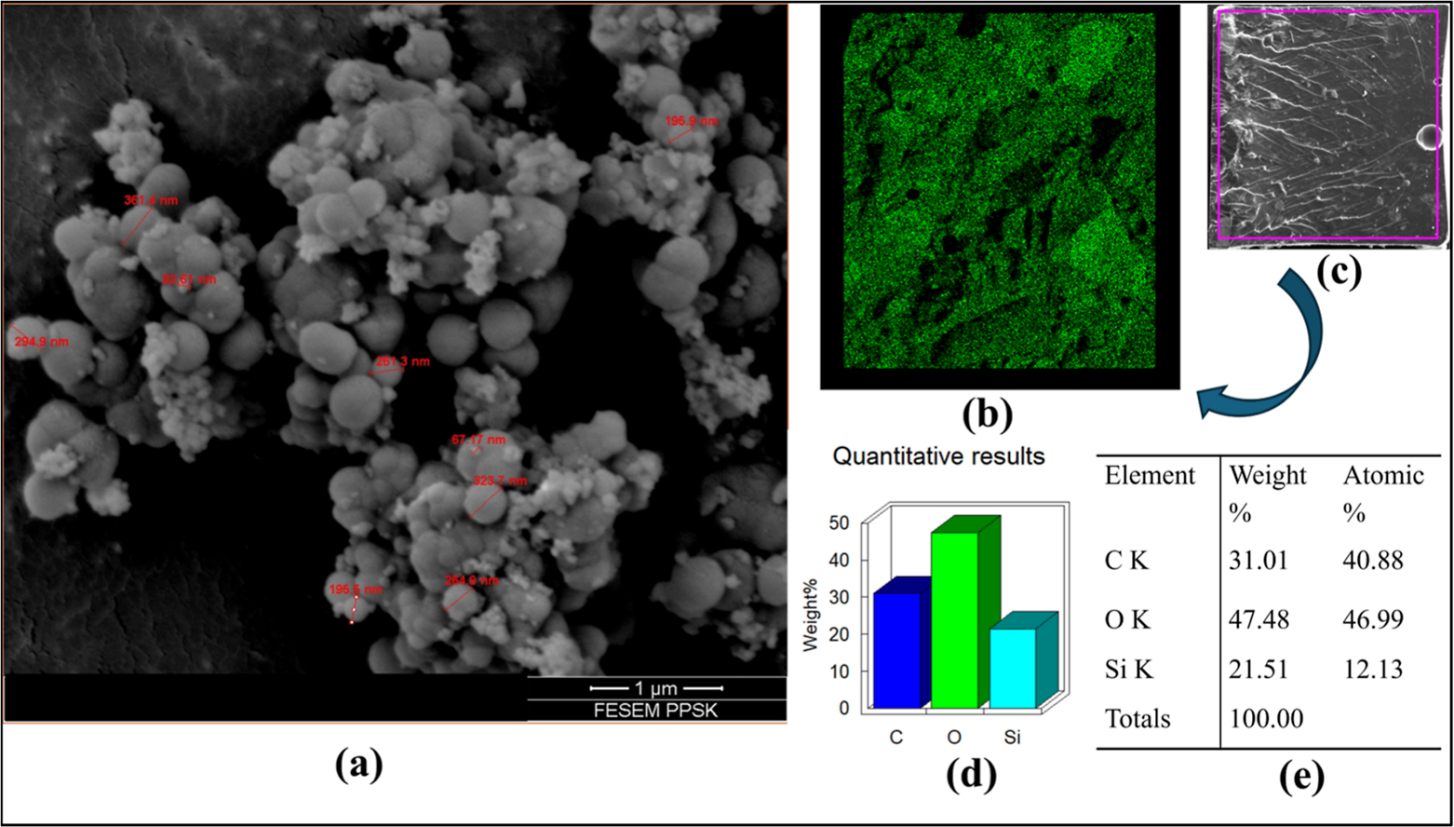
Characteristics of silica filler synthesized from TEOS. (a) Field Emission Scanning Electron Microscopy (FESEM) imaging with 50 k magnification. (b–e) Silica filler composition analysis by EDX. EDX analysis was performed using the same system to identify the elemental composition of the samples as in Section 2.4.3.

### 2.2 Synthesis of DAPEG 400

DAPEG 400 was synthesized by reacting PEG 400 (Sigma-Aldrich, USA) with acryloyl chloride (Sigma-Aldrich, USA) and triethylamine (Sigma-Aldrich, USA) (1:2:2 molar ratio) in dichloromethane (99.9%, Bendosen, Malaysia) under reflux overnight. The product was filtered using Whatman No. 1 filter paper (Whatman, UK) and purified using sodium hydrogen carbonate (NaHCO_3_, 99.14%, Bendosen, Malaysia) 10% hydrochloric acid (HCl, 37%, Merck, Germany) and distilled water, then dried with magnesium sulfate anhydrous (MgSO_4_, 99.2%, Bendosen, Malaysia) overnight. NMR spectra were recorded using a Bruker 300 MHz spectrometer (Bruker, Germany) with autosampler at 25 °C. Three groups of samples were dissolved in deuterated solvents: a deuterated chloroform (CDCl_3_). The acquired NMR data were processed using Topspin (version 4.4.1). All characteristics of DAPEG 400 are presented in Figure 2 as reported in a previous study.^17^

**Figure 2:**
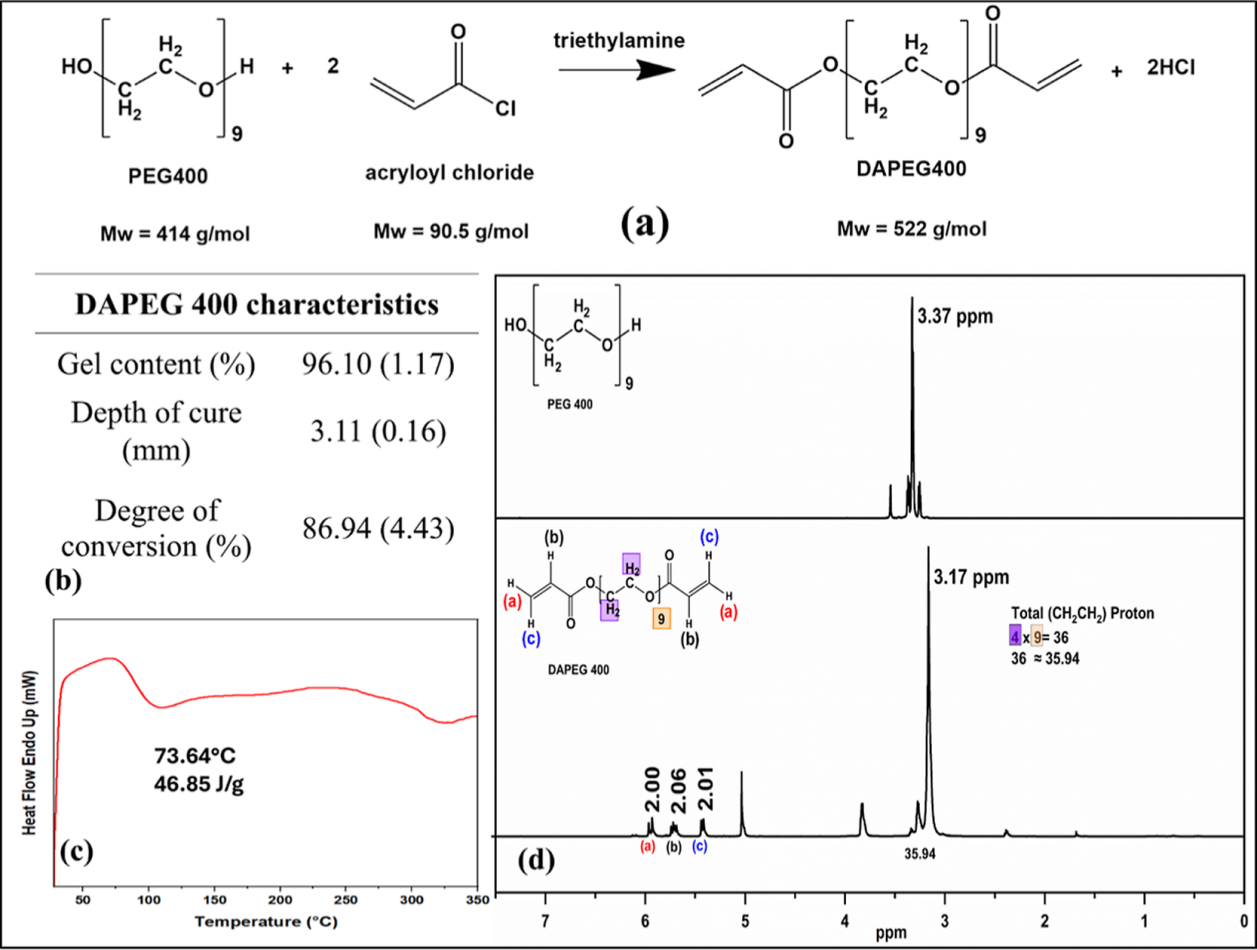
(a) DAPEG 400 synthesis, (b) DAPEG 400 characteristics, (c) DTG thermogram of DAPEG 400, (d) NMR spectra of DAPEG 400 (bottom) and its precursor PEG 400 (top).

### 2.3 Fabrication of DAPEG 400 Dental Composite Resin

The synthesized silica filler was incorporated into the diacrylated polyethylene glycol (DAPEG) resin matrix to produce composite formulations with varying filler loadings (Fi0–Fi60) as in Table 1. The appropriate amount of dried and ground silica was weighed and added to the DAPEG resin. Camphorquinone (97%, Sigma-Aldrich, USA), and 2-(Dimethylamino)­ethyl methacrylate (DMAEMA, 98%, Sigma-Aldrich, USA) were used as the photoinitiator and co-initiator, respectively. The composite was homogenized using a DK-300S II Speed Mixer (Kakuhunter, Japan) at 2000 rpm for 2 min. The resulting mixtures were then used for further fabrication and testing. The samples were subsequently photocured using a 200–005 BLUEDENT Smart built-in LED curing light unit (BG Light, Bulgaria) with a wavelength range of 430–490 nm, irradiance of 1200 mW/cm^2^, and a curing time of 60 s.

**Table 1:** Sample Preparation for Dental Composite Resin from DAPEG 400 with Silica Filler from TEOS

sample formulation abbreviation	filler loading (%)	resin DAPEG 400 (g)	silica filler (g)	photoinitiator (camphorquinone) (g)	Co-initiator (DMAEMA) (g)
Fi0	0	1	0	0.01	0.01
Fi10	10		0.11		
Fi20	20		0.25		
Fi30	30		0.43		
Fi40	40		0.67		
Fi50	50		1		
Fi60	60		1.5		

### 2.4 Characterization of Composites

#### 2.4.1 Chemical Characterization

##### 2.4.1.1 Fourier Transfer Infrared

The functional groups of the samples were analyzed using an FTIR spectrometer, Spectrum 100, (PerkinElmer, USA) which was calibrated by the manufacturer’s instructions. The sample was prepared by using potassium bromide (KBr) method followed by scanning. The identification of compounds was based on the changes in peak positions (functional groups) observed before and after the synthesis process. Three groups of FTIR spectra were recorded over a 4000–600 cm^–1^ wavelength range with 16 scans per sample in transmittance mode.

#### 2.4.2 Thermal Analysis

##### 2.4.2.1 Thermal Gravimetric Analysis

The Thermal Gravimetric Analysis (TGA) was conducted using the TGA/DSC 1 STAR System thermogravimetric analyzer (Mettler Toledo, USA). The sample was ground and weighed (3–11 mg). Four groups of different filler loadings were prepared, namely Fi0, Fi20, Fi40, and Fi60. The test was conducted at a heating rate of 10 °C/min in a nitrogen gas atmosphere, ranging from 30 to 800 °C. The results were obtained as TGA curves.

#### 2.4.3 Morphological Analysis

##### 2.4.3.1 Field Emission Scanning Electron Microscopy

The surface morphology and elemental composition of the samples were analyzed using field-emission scanning electron microscopy coupled with energy-dispersive X-ray spectroscopy (FESEM–EDX; FEI Quanta 450 FEG, FEI, Netherlands). Prior to analysis, the samples were sputter-coated with a thin layer of gold to enhance surface conductivity and minimize charging during imaging. FESEM analysis was conducted at an accelerating voltage of 15 kV and a working distance of 10 mm.

#### 2.4.4 Mechanical Properties

##### 2.4.4.1 Flexural Properties of Composites

Flexural strength was tested per ISO 4049 ^27^ using seven groups (*n* = 7) of bar-shaped samples (25 mm × 2 mm × 2 mm). Dimensions were measured with a digital calliper, and samples underwent a 3-point bending test (20 mm span) using a Shimadzu AG-XPlus machine at 1.0 mm/min with a 20 kN load cell. Flexural strength was calculated using eq 1.

1 $$\sigma =\frac{3Fl}{2bh^{2}}$$

where σ represents the flexural strength in megapascals (MPa), *F* is the maximum load in Newtons, *l* is the distance between the supports, *b* is the width, and *h* is the height of the specimen, both measured in millimeters immediately before testing.

##### 2.4.4.2 Compressive Properties of Composites

Compressive strength was tested per ISO 9917-2 ^28^ using seven groups (*n* = 7) of cylindrical samples (6 mm × 4 mm). Tests were conducted with a universal testing machine (Shimadzu AG-XPlus) at 1.0 mm/min using a 20 kN load cell. Strength was calculated using eq 2

2 $$Compressive\;strength=\frac{4p}{\pi d^{2}}$$

The compressive strength is calculated in megapascals (MPa), where *p* represents the maximum force applied in newtons and *d* is the measured diameter of the specimen in millimeters.

#### 2.4.5 Physical Properties

##### 2.4.5.1 Water Absorption of Composites

Water absorption was tested based on a modified ISO 4049 ^29^ method using seven groups (*n* = 7) of cylindrical samples (4 mm × 2 mm). The samples were dried for 24 h at 25 °C, then immersed in water for 2 months (60 days). Weight changes before and after immersion were used to calculate water absorption using eq 3.

3 $$Water\;absorption\left({\mu g/mm}^{3}\right)=\left(\frac{mf-m_{i}}{V_{0}}\right)\times 100$$

where *m*_*i*_ is the initial weight, in gramme (μg), of the sample before immersing in the water, *m*_*f*_ is the final weight, in gramme of the sample after immersing in the water, and *V*_0_ is the volume of sample in mm^3^.

##### 2.4.5.2 Volumetric Swelling of Composites

Volumetric swelling was tested based on a modified ISO 4049 ^29^ using seven groups (*n* = 7) of cylindrical samples (4 mm × 2 mm). After drying for 24 h, samples were immersed in water for 2 months (60 days). Volume was measured before and after immersion using a Mitutoyo digital micrometer, and dimension was calculated using eq 4.

4 $$Volumetric\;swelling\left(\%\right)=\left(\frac{V_{f}-V_{i}}{Vi}\right)\times 100$$

where *V*_*i*_ is the initial volume (mm) of the sample, before immersing in the water, and *I*_*f*_ is the final volume (mm) of the sample after immersing in the water.

##### 2.4.5.3 Polymerization Shrinkage of Composites

Polymerization shrinkage was measured at room temperature using a GR-200 balance (±0.0001 g) based on Archimedes’ principle (ISO 17304:2013 ^30^) Seven composite groups (Fi0–Fi60) with *n* = 7 were tested. Uncured and cured samples (6 mm × 4 mm) were weighed in air and in water to determine density changes. Shrinkage was calculated from the volume difference before and after polymerization using eq 5, reflecting the volumetric change from DAPEG 400 cross-linking.

5 $$Polymerization\;shrinkage\left(\%\right)=\frac{\rho_{c}-\rho_{u}}{\rho_{c}}\times 100$$

where polymerization shrinkage is in percentage (%), ρc is a density of cured resin, while ρu is the density of the uncured resin.

#### 2.4.6 Statistical Analysis

Data were analyzed using SPSS v28 with significance set at *p* ≤ 0.05. Descriptive statistics were reported as mean ± standard deviation (SD). Normality was assessed using the Shapiro–Wilk test, while homogeneity of variance was assessed using Levene’s test. For normally distributed data with homogeneous variance, one-way ANOVA followed by Tukey’s posthoc test was used to compare differences among groups. Graphs were generated using OriginPro 2024.

## 3 Results

### 3.1 Characterization of the DAPEG 400 Composite

#### 3.1.1 Fourier Transform Infrared (FTIR)

The FTIR spectra of the DAPEG 400 resin, silica filler, and composite materials with varying filler loadings are presented in Figure 3. The FTIR spectrum of the DAPEG 400 resin displays a prominent peak at 1723 cm^–1^, corresponding to the C=O stretching vibration indicative of the acrylate group. In contrast, the FTIR spectrum of the silica filler shows a strong peak at 1077 cm^–1^, associated with Si–O–Si stretching vibrations. For the composite materials with 50% silica filler loadings, the FTIR spectra revealed a combination of peaks from both the DAPEG 400 resin and silica filler. Notably, the composite with 50% silica loading exhibits peaks at both 1723 cm^–1^ and 1077 cm^–1^, indicating the presence of both acrylate and siloxane groups.^31–34^ The “fingerprint” region (1500–400 cm^–1^) for all three spectra could not be perfectly superimposed, indicating that they are not the same compounds, after all. These results are summarized in Figure 3A, highlighting the key peaks and their corresponding functional groups across different samples. The FTIR results indicate the successful synthesis and characterization of the DAPEG 400 composites.

**Figure 3:**
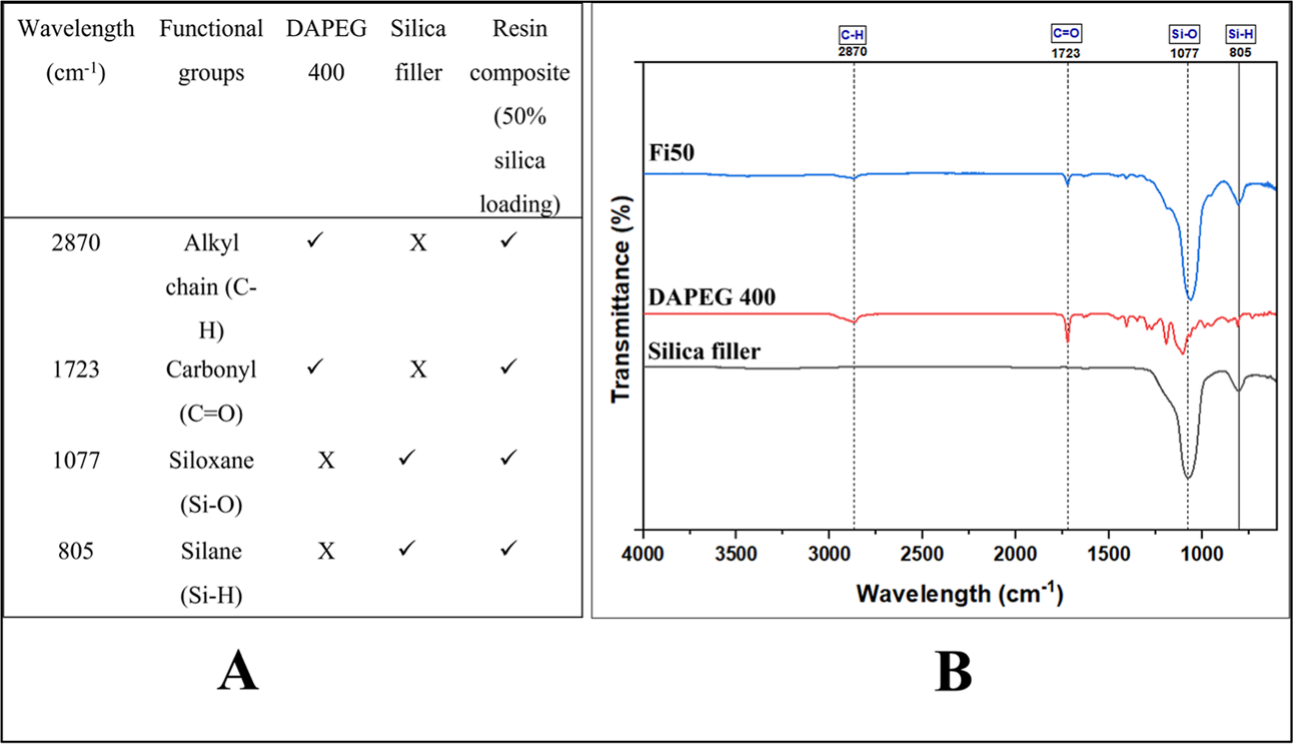
(A): Functional group, (B): FTIR spectra of DAPEG 400 composite with 50% filler loading (Fi50), DAPEG 400 monomer, and silica filler.

#### 3.1.2 Thermogravimetric Analysis of DAPEG 400 Composites

The thermal stability and decomposition behavior of cured DAPEG 400 resin and its composites with varying filler loadings were assessed using Thermogravimetric Analysis (TGA) and Differential Thermogravimetric (DTG) analysis. Although oral applications do not require thermal stability up to 800 °C, TGA confirms the material’s thermal resistance and filler presence, while DTG identifies degradation transitions. Figure 4 presents the compiled TGA and DTG results for DAPEG 400 composites with different filler loadings.

**Figure 4:**
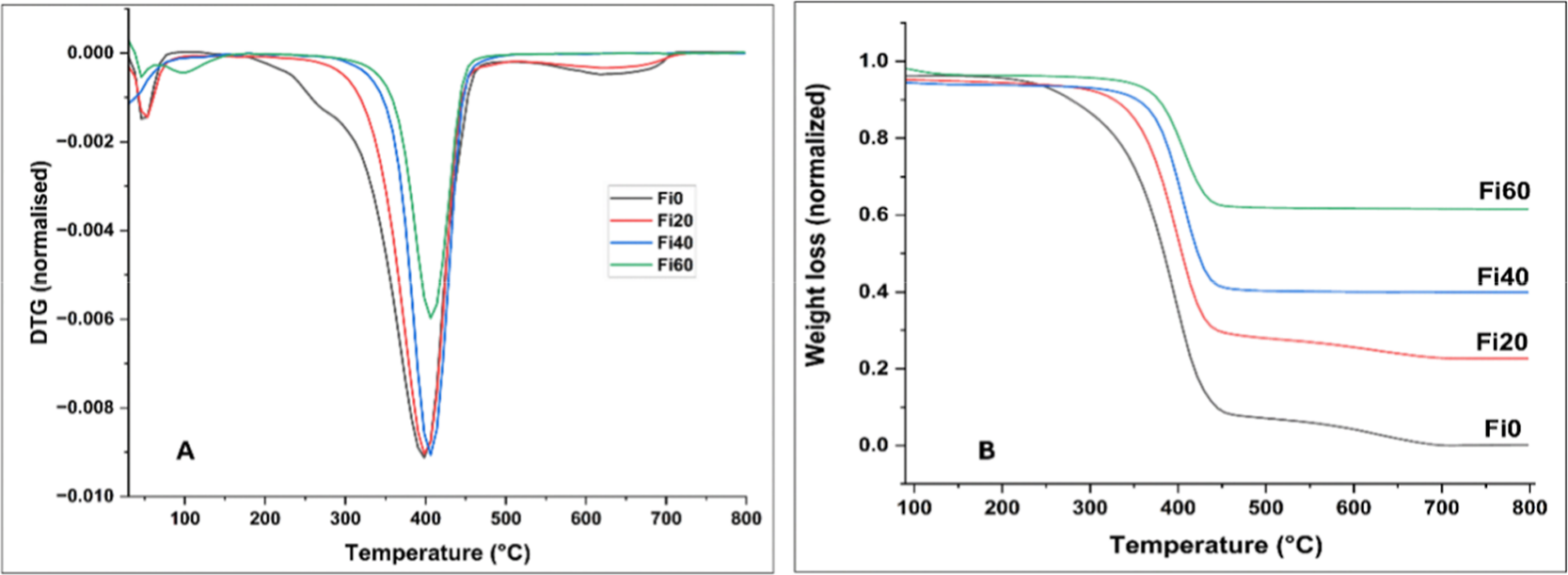
TGA & DTG compilation analysis of dental composite DAPEG 400 resins with different filler loading DTG compilation analysis of dental composite DAPEG 400 resins with different filler loading (A); TGA compilation analysis of dental composite DAPEG 400 resins with different filler loading (B).

DTG analysis (Figure 4A) confirms a lower degradation rate with silica, while increased residual mass indicates silica’s intact structure postdecomposition.^35^ This enhances durability, making the material ideal for heat-resistant applications like dental restorations.^36–38^ The TGA curves (Figure 4B) show that thermal degradation of neat DAPEG 400 begins around 300 °C, with complete decomposition by 400 °C. Adding silica filler shifts the degradation onset to higher temperatures and increases residual weight, indicating improved thermal stability. Residual weight at 800 °C corresponds to filler content (e.g., 22.95% for 20% filler), while Fi0 retains only 4.11% due to inorganic traces. Silica remains stable throughout, as its melting point is 1710 °C.

#### 3.1.3 Field Emission Scanning Electron Microscopy

The surface morphology of the DAPEG 400 composite resin with varying silica filler loadings (20%, 40%, and 60%) was analyzed using FESEM at magnifications of 1 k and 20 k. The FESEM images offer detailed insights into the dispersion and distribution of silica fillers within the resin matrix at each filler composition. Figure 5 shows the image analysis of FESEM for Fi20, Fi40, and Fi60, respectively, under 1 K and 20 K magnifications.

**Figure 5:**
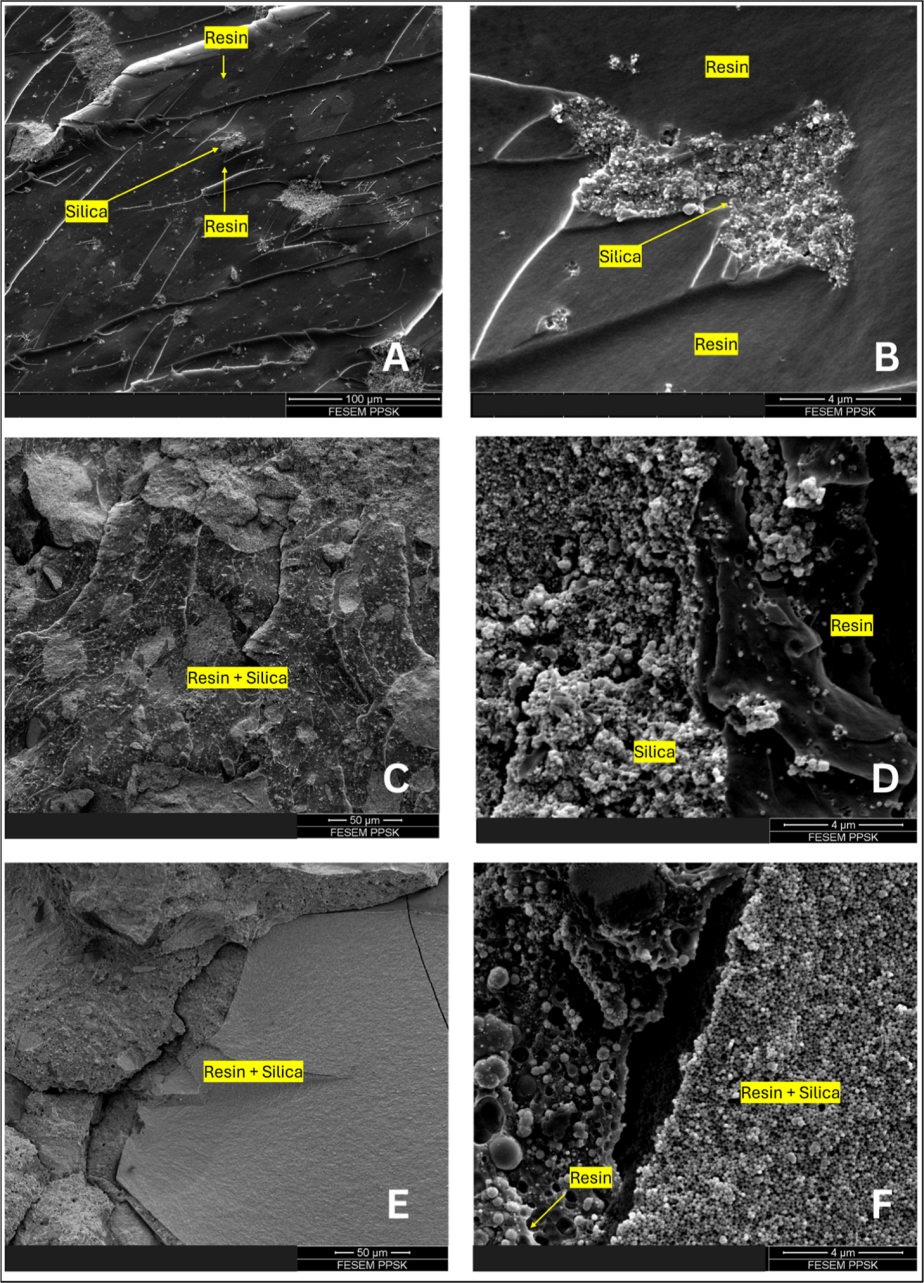
FESEM analysis of dental composite resin DAPEG 400 with Fi20, Fi40, and Fi60 filler loadings. (A) Dental composite resin DAPEG 400 with Fi20 Under 1 K Magnification; (B) dental composite resin DAPEG 400 with Fi20 under 20 k Magnification; (C) dental composite resin DAPEG 400 with Fi40 Under 1 K Magnification; (D) dental composite resin DAPEG 400 with Fi40 under 20 k Magnification; (E) dental composite resin DAPEG 400 with Fi60 under 1 k Magnification; (F) dental composite resin DAPEG 400 with Fi60 under 20 k Magnification.

At 1 k magnification, the FESEM image of the DAPEG 400 composite with the 20% silica filler shows dispersed particles with minimal agglomeration, indicating an overall uniform distribution within the resin matrix. As the filler loading increases to 40%, a denser and more uniformly dispersed silica structure is observed. At 60% filler loading, however, increased agglomeration is evident, suggesting that achieving a uniform distribution becomes challenging at higher filler concentrations. At 20 k magnification, these trends persist, with well-dispersed particles at lower filler loadings and more pronounced particle accumulation at 60%.

### 3.2 Mechanical Properties of the DAPEG 400 Composite

Before group comparison analysis, the assumptions for parametric testing were assessed. The Shapiro–Wilk test indicated that the data were normally distributed, and Levene’s test confirmed homogeneity of variance for the tested parameters (*p* > 0.05). Therefore, one-way ANOVA followed by Tukey’s posthoc test was applied.

#### 3.2.1 Flexural Strength

Flexural properties, particularly flexural strength, are fundamental indicators of a material’s capacity to resist deformation under applied loads.^39^ Figure 6 shows the flexural strength of the low-shrinkage dental composite resin. The results demonstrated a strong positive correlation between filler content and flexural strength. The unfilled composite (Fi0) showed the lowest strength at 3.40 MPa (SD = 0.71), while the highest filler content (Fi60) achieved 10.27 MPa (SD = 0.96). One-way ANOVA confirmed the differences were statistically significant (*F* = 107.82, *p* < 0.01), indicating that increasing silica filler significantly enhances the mechanical performance of the composite.

**Figure 6:**
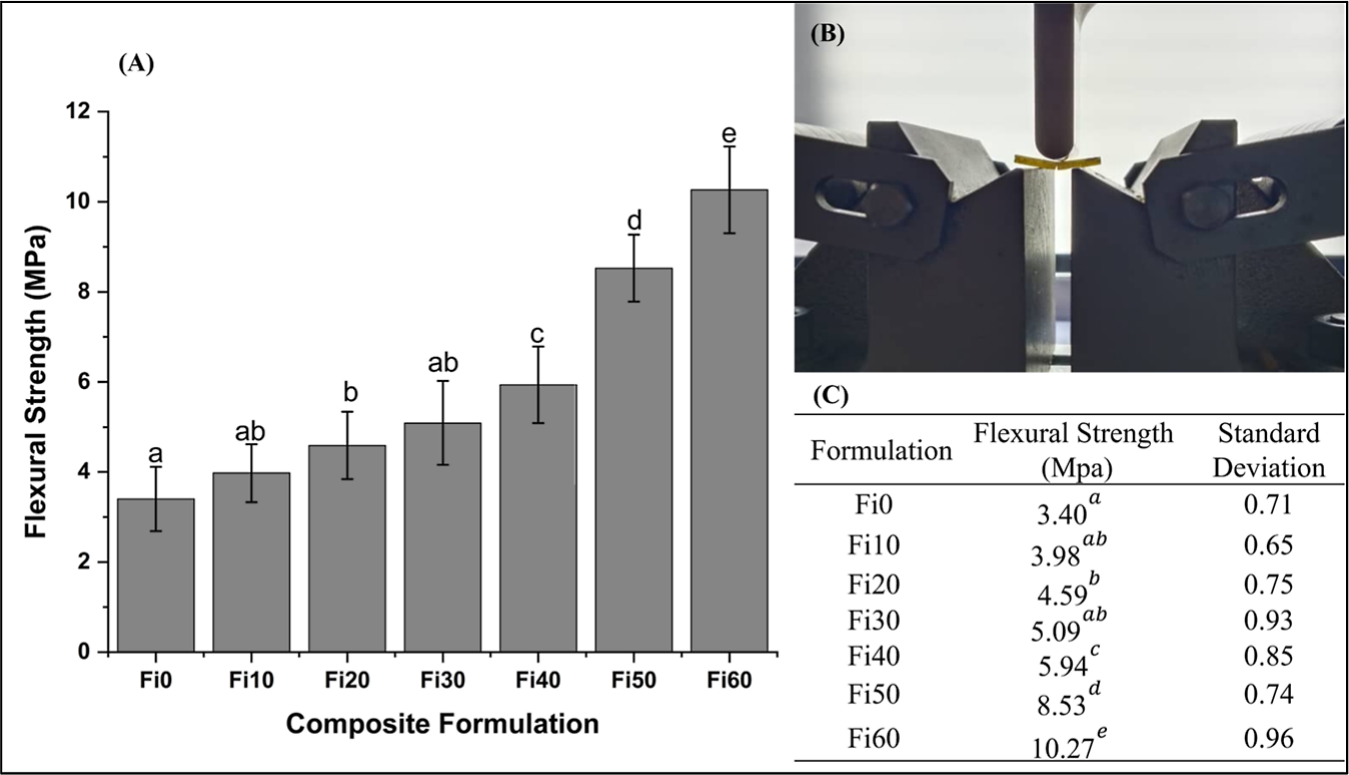
(A) Flexural strength of low-shrinkage dental composite DAPEG 400 with different filler loadings; (B) three-point bending test setup; (C) flexural strength with SD value. Statistical analysis was performed using one-way ANOVA followed by Tukey’s posthoc test. Statistical significance was set at *p* < 0.05. Different lowercase letters above the bars indicate statistically significant differences among groups. Groups sharing at least one letter are not significantly different. Data are presented as mean ± SD, *n* = 7.

#### 3.2.2 Compressive Properties

This study examined the impact of varying silica filler loadings on the compressive strength and modulus of DAPEG 400 low-shrinkage dental composites. The results highlight a strong correlation between an increasing filler content and improved compressive properties, which are essential for meeting the mechanical demands of dental applications. As shown in Figure 7, both compressive strength and modulus increased significantly with higher filler loadings. The compressive strength and modulus of the dental composite resin showed a strong dependence on the silica filler loading. One-way ANOVA confirmed significant effects on both compressive strength (*F* = 248.56, *p* < 0.01) and modulus (*F* = 267.28, *p* < 0.001). The unfilled composite (Fi0) had the lowest compressive strength at 3.36 MPa (SD = 0.95), while the highest strength was recorded at 60% filler loading (Fi60) with 41.42 MPa (SD = 2.80). Similarly, the compressive modulus increased significantly from 26.06 MPa (SD = 4.96) at Fi0 to 267.76 MPa (SD = 19.46) at Fi60, reflecting a significant enhancement in stiffness with an increasing filler content.

**Figure 7:**
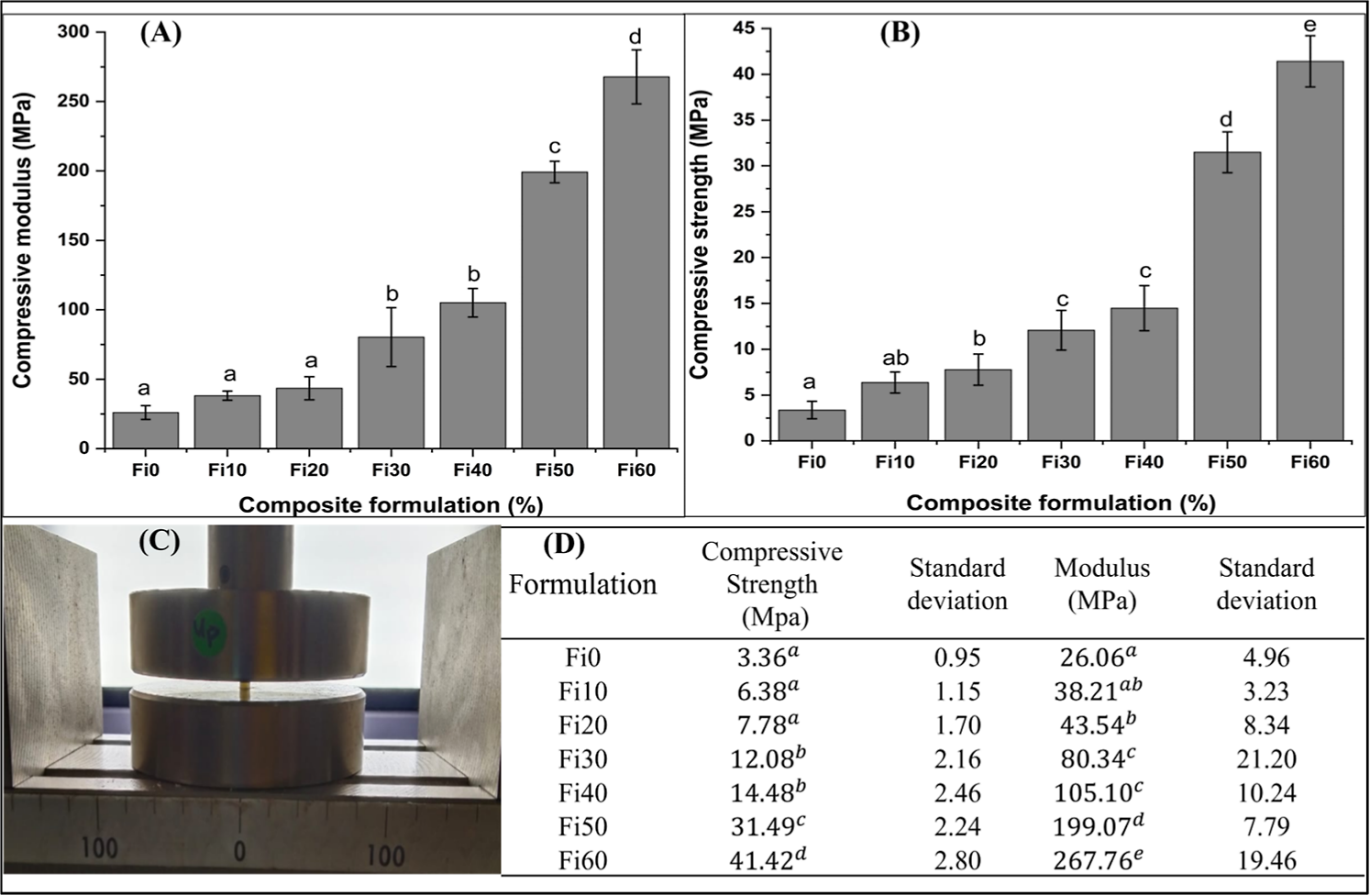
Compressive properties of the DAPEG 400 low-shrinkage dental composite with different filler loadings: (A) compressive strength; (B) composite modulus; (C) compressive test setup; (D) compressive properties with SD value. Statistical analysis was performed using one-way ANOVA followed by Tukey’s posthoc test. Statistical significance was set at *p* < 0.05. Different lowercase letters above the bars indicate statistically significant differences among groups. Groups sharing at least one letter are not significantly different. Data are presented as mean ± SD, *n* = 7.

#### 3.2.3 Water Absorption and Volumetric Swelling

The water absorption and volumetric swelling of DAPEG 400-based dental composites were significantly affected by silica filler loading (Fi0–Fi60), as shown in Figure 8. DAPEG 400, a hydrogel monomer, showed the highest water absorption at 0% filler (Fi0) with 777.57 μg/mm^3^ (SD = 60.00). As filler content increased, water absorption decreased, reaching 481.68 μg/mm^3^ at Fi30 (SD = 22.52), with slight fluctuations at higher loading, rising slightly to 538.86 μg/mm^3^ (SD 20.58) at Fi40, then decreasing to 430.29 μg/mm^3^ (SD = 12.27) at Fi50 and 475.32 μg/mm^3^ (SD = 22.76) at Fi60. These variations may relate to silica’s surface interactions, which adsorb water without absorbing it into the structure. ANOVA confirmed these differences were statistically significant (*F* = 73.369, *p* < 0.001). Similarly, volumetric swelling declined with increased filler loading. The highest swelling was observed at Fi0 (98.30%, SD = 4.77), decreasing steadily to 33.37% (SD = 2.26) at Fi60. The reduction is attributed to the rigid silica filler, which restricts expansion of the hydrophilic matrix. This trend was also statistically significant (*F* = 85.312, *p* < 0.001), indicating a statistically significant effect of filler content on dimensional stability.

**Figure 8:**
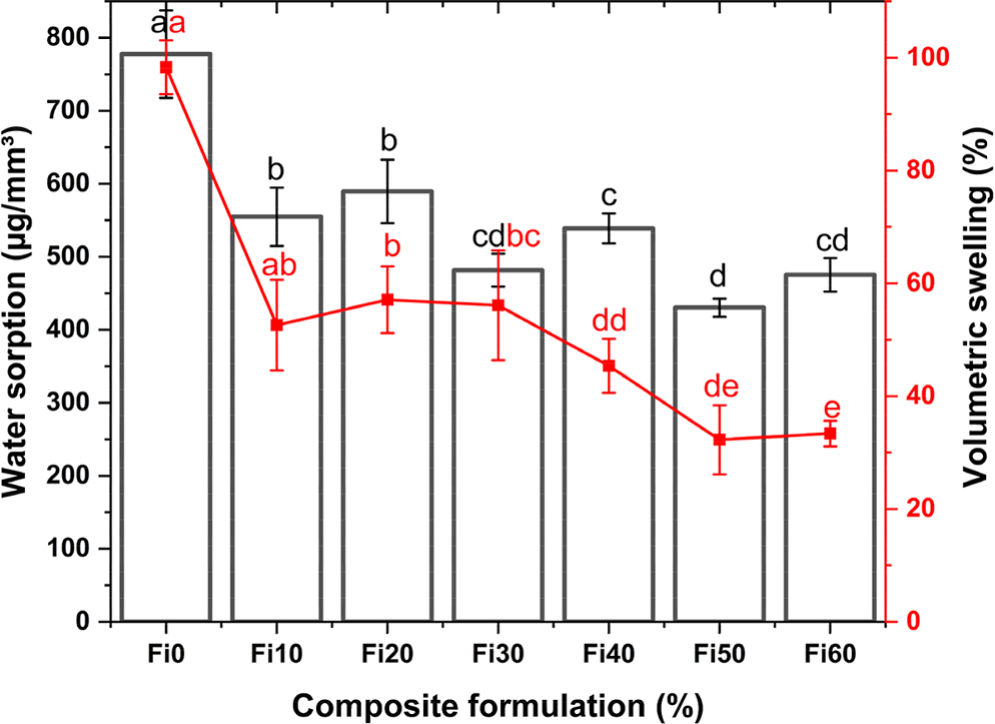
Water absorption and volumetric swelling of the DAPEG 400 low-shrinkage composite with different filler loadings. Statistical analysis was performed using one-way ANOVA followed by Tukey’s posthoc test. Statistical significance was set at *p* < 0.05. Different lowercase letters above the bars or data points indicate statistically significant differences among groups. Groups sharing at least one letter are not significantly different. Data are presented as mean ± SD, *n* = 7.

#### 3.2.4 Polymerization Shrinkage of DAPEG 400 Composites

The polymerization shrinkage of the dental composite resin decreased consistently with increasing silica filler loading, as the fillers themselves do not undergo shrinkage. As shown in Figure 9, the composite without filler (Fi0) had the highest shrinkage at 7.87% (SD = 0.52). This value gradually declined with each increase in the filler content: 7.48% (Fi10, SD = 0.24), 5.38% (Fi20, SD = 0.46), 4.67% (Fi30, SD = 0.35), 3.41% (Fi40, SD = 0.84), and 2.91% (Fi50, SD = 0.54). The lowest shrinkage was recorded at 60% filler loading (Fi60), with a value of 1.58% (SD = 0.24). This value indicates that increasing the silica filler loading reduced shrinkage within the experimental DAPEG 400 composite system. However, direct comparison with commercial low-shrinkage dental composites was not performed in this study; therefore, the results should be interpreted as an in vitro finding within the tested formulations rather than evidence of superiority over commercial materials.

**Figure 9:**
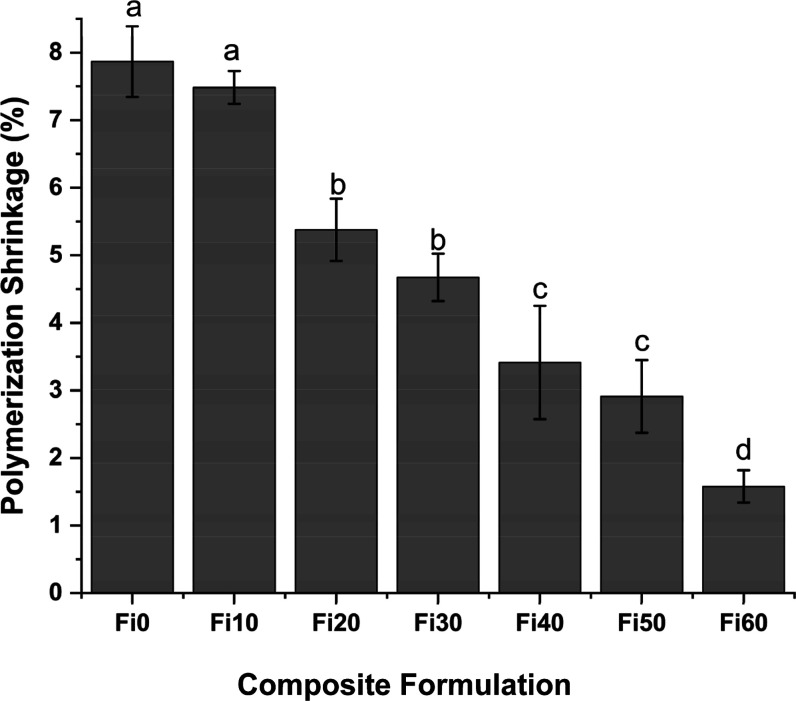
Polymerization shrinkage of the DAPEG 400 low-shrinkage dental composite with different filler loadings. Statistical analysis was performed using one-way ANOVA followed by Tukey’s posthoc test. Statistical significance was set at *p* < 0.05. Different lowercase letters above the bars indicate statistically significant differences among groups. Groups sharing at least one letter are not significantly different. Data are presented as mean ± SD, *n* = 7.

## 4 Discussion

For the characterization of the low-shrinkage dental composite, the presence of the peak at 1723 cm^–1^ in all composite samples confirms the effective incorporation of the DAPEG 400 resin, while the consistent appearance of the peak at 1077 cm^–1^ across composites with different filler loadings suggests a well-dispersed silica filler within the resin matrix as shown in FESEM analysis. The thermal stability of cured DAPEG 400 resin shows an improvement with silica filler addition, as shown by TGA and DTG analyses.^40^ Silica acts as a heat barrier, delaying and reducing the rate of degradation.^41^ In neat DAPEG 400, degradation occurs rapidly due to its organic nature,^42^ but silica’s high thermal resistance slows down this process. Higher filler content forms a silica network that restricts polymer chain mobility and distributes heat, further stabilizing the composite.^43^ The polymerization efficiency of the pure DAPEG system has been previously reported, demonstrating a high degree of conversion (∼86%) and gel content (∼96%).^17^ These values are comparable to or higher than those typically reported for conventional methacrylate-based dental composites, which generally exhibit degree of conversion values in the range of approximately 55–75%, depending on formulation and curing conditions. This suggests that the incorporation of the hydrogel-based monomer does not compromise polymerization efficiency.

For mechanical properties, the flexural strength shows the highest value with a peak at 60% filler loading. This improvement is due to silica particles distributing stress evenly and reinforcing the resin matrix. The improvement in mechanical performance can be attributed to the stress-transfer mechanism, where silica particles distribute mechanical forces more evenly, preventing localized stress accumulation and delaying crack propagation.^44^ Additionally, strong interfacial adhesion between the silica filler and the resin matrix enhances load transfer efficiency, while the reduction in polymerization shrinkage at higher filler loadings improves structural integrity.^45^ However, excessive filler content could lead to brittleness and reduced handling efficiency, necessitating a balance between mechanical reinforcement and workability.^46,47^ The findings align with previous studies, indicating that optimized filler distribution enhances mechanical strength up to a threshold, beyond which excessive filler may cause agglomeration and weaken the composite.^48,49^

Dental restorations, particularly in posterior teeth, must endure constant compressive stresses due to biting and chewing forces.^50^ This resistance to compressive forces is essential for the long-term durability of composite restorations, allowing the material to mimic the mechanical behavior of natural teeth and provide both functionality and longevity in clinical applications.^51^ The unfilled composite (Fi0) displayed the lowest compressive strength, while the addition of silica fillers significantly improved the performance. The most statistically significant improvement was observed at higher filler loadings, wherein compressive strength increased with increasing silica filler loading between 40% and 60% filler content.^52,53^ Similarly, the compressive modulus increased significantly with increasing filler content, reaching its highest value at 60% filler loading.^24^ Fillers in the range of 40%–60% offer an optimal balance between strength and rigidity, making the material suitable for high-stress applications such as posterior restorations. However, excessive filler content may increase brittleness, potentially compromising fracture resistance.^54^ Therefore, an optimal filler loading range is necessary to maximize the mechanical performance without sacrificing workability or resilience.

The enhancement in mechanical properties is strongly influenced not only by filler loading but also by the dispersion quality of the silica particles and the interfacial interactions between filler and matrix. FESEM observations indicate relatively uniform dispersion at lower to intermediate filler loadings, which promotes efficient stress transfer and reduces localized stress concentrations within the composite. This uniform distribution allows applied loads to be effectively distributed across the matrix–filler network, thereby improving both strength and stiffness.^55^ The presence of ether linkages (–C–O–C–) in the PEG backbone enhances interaction with the silica surface, potentially through hydrogen bonding or polar interactions, leading to improved adhesion at the interface.^56^ This strong interfacial interaction facilitates effective load transfer from the matrix to the filler particles, contributing to the observed mechanical reinforcement.^57,58^ However, at higher filler loading (Fi60), the onset of particle agglomeration observed in FESEM images may reduce dispersion uniformity, which can introduce stress concentration points and limit further mechanical improvement.

The dimensional stability of DAPEG 400 composites is influenced by the hydrophilic PEG backbone, hydrophobic acrylate end groups, and silica filler content. At low filler loading (Fi0), high water absorption occurs due to PEG’s hydrophilicity,^59^ while increased filler content reduces absorption by limiting resin-water interaction.^60^ At high filler levels (Fi40, Fi60), slight absorption increases a numerical increase in water absorption was observed due to silica’s water adsorption.^61^ Volumetric swelling follows this trend, highest at Fi0 and decreasing with more filler, as rigid silica limits expansion.^62^ The hydrophobic acrylate end groups further enhance stability.^63^ Polymerization shrinkage is mitigated by water uptake from PEG and restricted by silica fillers, reducing stress.^64,65^ Clinically, higher filler loading minimizes water absorption, swelling, and shrinkage, preserving marginal integrity and restoration durability. Our composite had the average water absorption of 475.32 μg/mm^3^ (SD = 22.76) at Fi60 about 13 times more than the average of existing dental materials (36.11 μgram/mm^3^,.^66,67^ The minimum water absorption specified by ISO 4049 for dental restorative materials but luting materials is 40 μgram/mm^3^. The idea behind ISO 4049’s limitation value of water absorption was due to the concern of plasticization (softening) of dental composites, leaching out of unpolymerized monomers, and staining. However, in this work, water absorption is used as a tool to eradicate shrinkage with no negative consequences. The composite samples demonstrated very low shrinkage (1.58%, SD = 0.24), while still intact even after 60 days of water immersion, with minimum leaching (96.10% gel content), and no staining.

The relationships among water absorption, volumetric swelling, and dimensional stability in the present system differ from those of conventional dental composites. While excessive water uptake is generally undesirable, the hydrogel-based nature of the DAPEG matrix allows for controlled water absorption to serve a functional role. The absorbed water induces limited expansion of the polymer network, which partially offsets the volumetric contraction caused by polymerization shrinkage.^68,69^ As silica filler loading increases, both water absorption and volumetric swelling decrease, indicating that the rigid filler phase effectively restricts excessive expansion of the hydrophilic matrix.^70^ This controlled behavior ensures that expansion remains within a beneficial range, avoiding structural deformation while still contributing to shrinkage compensation.

The reduction in shrinkage with higher filler loadings occurs as fillers displace the resin matrix, the main contributor to shrinkage.^71^ A lower filler content results in greater shrinkage due to a higher proportion of reactive monomers.^72,73^ Increased filler content restricts polymer chain mobility, minimizing contraction, with 60% filler loading significantly reducing shrinkage.^74^ Clinically, lower shrinkage composites improve marginal integrity, reducing microleakage and failure risks.^75,76^ An optimal balance is crucial to maintaining viscosity, handling, and wear resistance.^77^ Polymerization shrinkage in conventional methacrylate-based systems arises from the conversion of van der Waals interactions between monomer molecules into shorter covalent bonds during cross-linking, resulting in volumetric contraction and the generation of internal stress.^78^ In the present system, this effect is mitigated through the incorporation of a hydrophilic DAPEG hydrogel monomer that introduces a complementary expansion mechanism.

Following polymerization, the PEG-based network undergoes controlled water uptake due to its hydrophilic polyether backbone.^79^ This water absorption induces volumetric expansion, which partially offsets the initial polymerization contraction. As a result, the net dimensional change is significantly reduced, as reflected by the low shrinkage values observed at higher filler loadings. Importantly, this shrinkage compensation mechanism contributes to reducing interfacial stress at the tooth composite interface. Lower contraction stress minimizes the risk of marginal gap formation, microleakage, and debonding, which are common causes of restoration failure.^80^ In addition, the presence of a silica filler regulates this expansion by restricting excessive swelling, thereby maintaining structural integrity and preventing crack formation.^81^ This relation between polymerization contraction and hydrogel-induced expansion represents a novel strategy for improving the dimensional stability while preserving interfacial bonding performance in dental composite systems.

The influence of silica filler loading on the performance of the composite system follows a clear and systematic trend. As filler content increases, mechanical properties such as flexural strength, compressive strength, and modulus improve significantly. This enhancement is attributed to the reinforcing effect of silica particles, which facilitate efficient stress transfer and increase the overall stiffness of the composite. Previous studies have shown that strong filler–matrix interactions, particularly in systems with surface-functionalized silica, can further enhance load transfer efficiency and mechanical performance.^82^ Simultaneously, polymerization shrinkage decreases progressively with increasing filler loading. This is primarily due to the reduction in the volume fraction of the resin matrix, which is the main contributor to shrinkage.^83^ The incorporation of silica filler effectively dilutes the polymerizable components, thereby limiting volumetric contraction during curing. At higher filler loading (e.g., Fi60), the composite achieves an optimal balance between mechanical reinforcement and shrinkage reduction. However, excessive filler content may lead to particle agglomeration, as observed in FESEM analysis, which can reduce dispersion uniformity and introduce structural heterogeneities that negatively affect mechanical performance. Similar behavior has been reported in polymer composites, where optimal filler loading enhances properties, while excessive filler leads to nonuniform structures and diminished performance.^23,84^ A major limitation of this study is the absence of a commercial control material tested under the same experimental conditions. Although the polymerization shrinkage, water absorption, dimensional stability, and mechanical properties of the DAPEG 400 composites were discussed in relation to values reported in the literature, such comparisons should be interpreted cautiously. These properties are strongly influenced by specimen geometry, curing protocol, filler composition, storage conditions, immersion duration, and testing method. Therefore, the present findings demonstrate the behavior of the experimental DAPEG 400 composite system only. Future studies should include representative conventional and low-shrinkage commercial dental composites tested under identical conditions to allow direct performance comparison.

## 5 Conclusion

A low-shrinkage composite resin (1.58%, SD = 0.24) was successfully developed using a DAPEG 400 hydrogel monomer reinforced with a 60% silica filler. The expandable nature of the resin, combined with the nonshrinking filler, improved the selected physicomechanical properties and reduced polymerization shrinkage within the experimental formulations, with Fi60 showing the lowest shrinkage value among the tested groups. The findings suggest that the combination of an expandable DAPEG 400 matrix and a silica filler may contribute to shrinkage compensation and dimensional stability under in vitro conditions. However, this study did not include a direct comparison with commercial restorative composites, bonding evaluation, wear resistance testing, long-term durability assessment, or biocompatibility analysis. Therefore, further standardized investigations are required before any conclusions regarding clinical applicability can be made.
